# Supplementary material for: Effects of Gastrodin against Lead-Induced Brain Injury in Mice Associated with the Wnt/Nrf2 Pathway
Source: Nutrients. 2020 Jun 17;12(6):1805. doi: 10.3390/nu12061805 (PMC7353406; doi:10.3390/nu12061805)
Supplement: Supplementary file 1 [file nutrients-12-01805-s001.pdf]

Table S1. Effects of gastrodin (GAS) on learning and memory abilities in lead-exposed mice in the step-down test (Complementary material).

Learning training test latency (second)

Control	Pb	Pb+GAS(50mg/kg)	Pb+GAS(100mg/kg)
92.31±6.21	53.85±7.92	42.36±6.03	82.98±10.76
90.14±9.62	38.54±9.52	69.57±6.87	90.57±9.53
76.07±7.34	57.18±10.24	58.71±8.23	102.43±11.12
80.31±9.19	63.63±7.78	67.28±10.35	83.62±7.13
64.37±12.12	58.74±14.82	75.32±11.03	110.82±8.89
72.68±11.12	49.07±6.98	78.05±9.32	87.86±8.25
68.82±7.43	69.14±9.91	82.89±7.34	91.48±10.37
90.43±5.15	53.73±13.85	72.79±7.33	79.83±9.92
59.55±11.13	66.54±8.36	68.03±6.24	100.82±8.73
86.06±5.62	38.79±12.41	81.72±10.03	93.15±11.73
87.36±13.01	49.39±7.37	61.43±7.51	91.27±8.43
86.83±7.72	56.03±9.79	69.27±8.31	84.26±11.58
86.37±6.56	68.19±14.53	74.93±6.41	83.13±7.82
88.24±5.16	37.74±11.96	80.47±7.56	79.72±10.73
86.58±11.48	55.53±9.67	69.56±11.04	81.23±9.86

Memory test latency (second).

Control	Pb	Pb+GAS(50mg/kg)	Pb+GAS(100mg/kg)
95.12±12.42	56.76±10.13	59.16±9.76	84.19±6.83
116.24±11.12	68.82±8.71	79.25±8.44	92.58±8.17
106.14±8.24	51.18±7.82	67.23±9.85	83.17±11.32
113.56±12.25	73.97±11.85	87.13±11.28	76.22±9.24
107.42±10.62	63.34±9.96	85.44±9.57	85.03±7.62
121.75±11.31	72.65±8.76	90.52±9.35	69.04±9.64
87.63±11.24	67.86±11.16	84.09±8.92	84.49±10.36
138.04±9.82	89.49±10.25	90.25±8.73	85.72±9.06
112.28±13.91	68.53±7.84	82.86±9.58	76.32±7.94
95.02±10.08	49.78±9.16	91.12±9.84	82.25±8.37
108.31±14.73	73.32±7.23	88.76±11.57	81.43±6.67
98.82±8.89	67.93±8.07	90.37±9.82	78.54±8.86
110.31±10.43	68.19±10.14	87.02±9.87	58.53±7.62
76.52±11.73	72.15±9.72	86.17±12.93	65.31±10.81
99.67±9.78	63.02±8.12	79.14±9.92	84.93±9.67

The number of errors in learning training test.

Control	Pb	Pb+GAS(50mg/kg)	Pb+GAS(100mg/kg)
1.75±0.18	4.91±0.63	3.59±0.35	2.82±0.66
2.62±0.27	4.39±0.54	3.75±0.24	2.35±0.29
2.91±0.28	3.87±0.43	2.98±0.59	2.74±0.53
1.56±0.49	4.64±0.37	3.83±0.33	2.62±0.45
3.02±0.36	3.85±0.56	2.74±0.37	1.97±0.37
1.81±0.31	4.72±0.42	4.82±0.35	2.84±0.46
2.73±0.42	3.94±0.36	3.89±0.32	3.48±0.32
2.04±0.62	4.89±0.51	3.65±0.31	4.23±0.39
2.52±0.46	4.97±0.64	3.86±0.28	3.72±0.37
2.94±0.27	3.81±0.49	3.93±0.56	3.45±0.41
2.32±0.33	3.67±0.33	3.89±0.34	3.67±0.63
3.02±0.36	4.76±0.67	2.99±0.22	2.52±0.52
2.32±0.32	3.95±0.38	3.86±0.37	3.13±0.42
2.73±0.52	4.93±0.53	2.97±0.53	2.62±0.37
2.62±0.73	3.98±0.14	3.68±0.52	2.23±0.59

The number of errors in memory test.

**Control**	**Pb**	**Pb+GAS(50mg/kg)**	Pb+GAS(100mg/kg)
0.87±0.11	2.53±0.42	1.16±0.12	0.87±0.13
0.68±0.12	2.38±0.32	1.23±0.17	0.93±0.17
0.78±0.05	2.17±0.24	1.16±0.07	1.17±0.21
0.73±0.19	1.63±0.38	1.21±0.12	0.73±0.14
0.43±0.12	2.54±0.48	1.22±0.08	1.15±0.11
0.91±0.11	2.97±0.29	2.01±0.13	0.62±0.13
0.83±0.15	2.64±0.19	1.22±0.14	0.83±0.16
0.52±0.06	2.33±0.35	1.04±0.12	1.17±0.21
0.95±0.13	2.24±0.36	1.13±0.09	0.86±0.14
0.76±0.07	2.32±0.41	1.12±0.14	0.82±0.12
0.83±0.09	2.43±0.37	1.03±0.11	1.03±0.12
0.78±0.07	2.56±0.27	1.22±0.16	0.87±0.09
0.69±0.06	2.19±0.56	1.21±0.13	1.25±0.12
0.72±0.11	2.37±0.27	1.23±0.12	0.95±0.14
0.79±0.08	1.53±0.23	0.96±0.07	0.92±0.15
